# Supplementary material for: Antitumor Activity and Mechanism of Action of Hormonotoxin, an LHRH Analog Conjugated to Dermaseptin-B2, a Multifunctional Antimicrobial Peptide
Source: Int J Mol Sci. 2021 Oct 20;22(21):11303. doi: 10.3390/ijms222111303 (PMC8582938; doi:10.3390/ijms222111303)
Supplement: Supplementary file 1 [file ijms-22-11303-s001.zip › ijms-1402026-supplementary.pdf]

SUPPLEMENTARY DATA

Antitumor activity and mechanism of action of the Hormonotoxin, an LHRH analog conjugated to Dermaseptin-B2, a multifunctional antimicrobial peptide.

Mickael Couty[1], Marie Dusaud[1¶], Mickael Miro-Padovani[1¶], Liuhui Zhang[1¶], Patricia Zadigue[1], Loussiné Zargarian[2], Olivier Lequin[3], Alexandre De La Taille[1], Jean Delbe[1], Yamina Hamma-Kourbali[1, &] and Mohamed Amiche[1, 4&*].

1. Université Paris Est Créteil, INSERM, IMRB, F-94010 Créteil, France ; mickael.couty@inserm.fr (MA); marie.dussaud@gmail.com (MD); mickael.miro-padovani@aphp.fr (MMP); quingfengxu0716@126.com (LZ); zadigue@gmail.com(PZ); adelataille@hotmail.com (ADLT); delbe@u-pec.fr (JD); hamma@u-pec.fr (YHK); mohamed.amiche@sorbonne-universite.fr (MA)

2. LBPA, CNRS UMR 8113 École Normale Supérieure Paris-Saclay, 4 Avenue des Sciences, 91190 Gif-sur-Yvette, France; loussine.zargarian@ens-cachan.fr (LZ)

3. Sorbonne Université, École Normale Supérieure, PSL University, CNRS, Laboratoire des Biomolécules (LBM), 75005 Paris, France : olivier.lequin@upmc.fr (OL)

4 Current Address : Sorbonne Université, CNRS, Institut de Biologie Paris-Seine, IBPS, BIOSIPE, F-75252 Paris, France : : mohamed.amiche@sorbonne-universite.fr (MA)

* Correspondence: mohamed.amiche@sorbonne-universite.fr (MA)

¶ These authors have contributed equally

& YHK and MA are joint senior authors

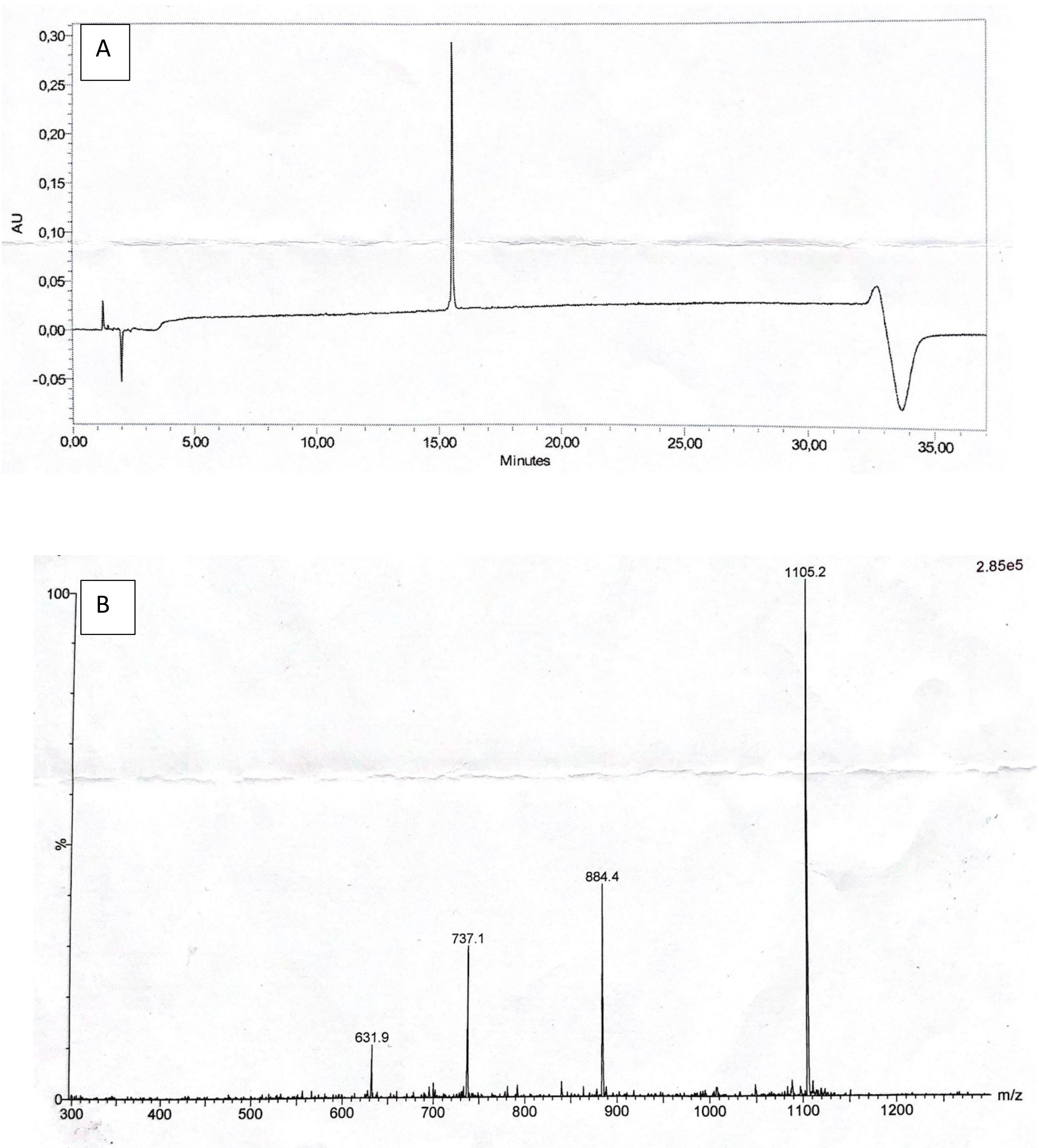

Figure S1. (**A**): RP-HPLC profile of synthetic H-B2. (**B**): ESI-MS spectra of synthetic H-B2.

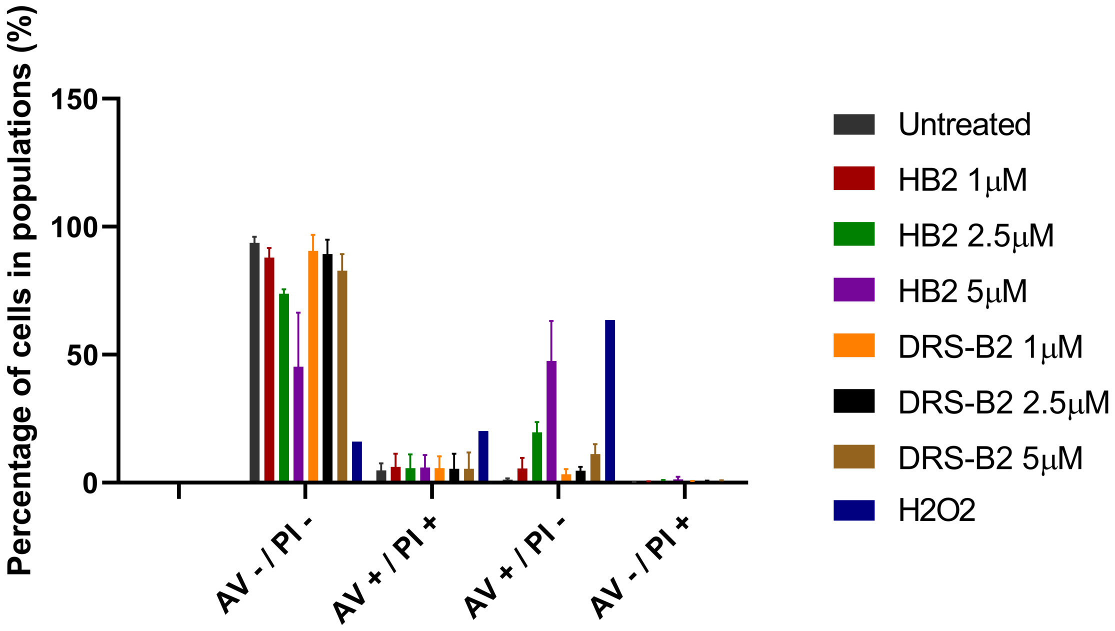

Figure S2: Histogram of each population obtained by flow cytometry in the different conditions (n=3).
